# Supplementary material for: Causal inference in ethnographic research: Refining explanations with abductive logic, strength of evidence assessments, and graphical models
Source: PLoS One. 2024 May 7;19(5):e0302857. doi: 10.1371/journal.pone.0302857 (PMC11075859; doi:10.1371/journal.pone.0302857)
Supplement: S1 Appendix — (DOCX) [file pone.0302857.s001.docx]

S1 Appendix. Causal Concepts Primer.

We start with concepts and vocabulary used by social scientists who consciously practice causal inference, an introduction that can formalize and offer language for ideas that anthropologists may use but not consciously thematize. The analysis of causality has a long history in Western philosophy (commonly traced to (Hume, 2000)), but practicing social scientists agree on basic criteria for empirical analysis of causes. To support claiming that some factor X causes some other, say Y: 1) X and Y must empirically be associated; 2) X must precede Y in time; and 3) No third factor can underlie both X and Y that causes them to be associated even though neither causes the other. (See Figure 1 for some common graphical representations.)


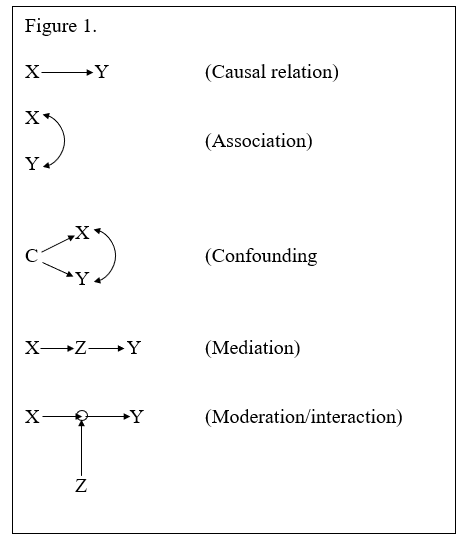


Thus, to show that consuming alcohol causes auto accidents, we first need evidence (perhaps but not necessarily statistical) that an association exists between drivers’ levels of alcohol consumption and accident incidence. Second, we need to show that the alcohol consumption was prior in time, precluding the possibility that having an accident caused alcohol consumption. That criterion is often described as ruling out “reverse causality,” where Y and X are associated because Y causes X. The third and crucial criterion involves ruling out that the association arises from a “confounding” variable C, as depicted in Figure 1. In the current example, someone might propose that gender is a confounder (C) that affects both alcohol use and accident propensity, creating an empirical association (curved double-headed arrow) between X and Y even though neither caused the other. Presuming for simplicity a two-gender culture: If men are socialized into X) heavier alcohol consumption and Y) accident-conducive driving habits, then gender socialization could create an association between X and Y even if alcohol use didn’t cause accidents. Thus, to fulfill this last criterion one must prove a negative, that is, somehow rule out the possibility that the X and Y are associated only because they share a common cause in the confounding variable “C.” Many statistical techniques exist for this purpose, and meaningful advances in statistical techniques to do this have occurred in recent decades. They all share examining what happens to the X-Y association when the purported confounder C is “controlled.” In the current example, the logic would involve examining the alcohol-accident association in samples containing just a single gender, under which the X-Y associations should vanish if C is a confounder. When possible, randomized controlled experiments are the ideal for controlling potential confounders, but that possibility rarely exists for the naturalistically observing ethnographer. Excluding confounders is challenging: Perhaps gender can be ruled out, but what about some other as other confounding such as “aggressive personality,” which might also cause alcohol and accident-prone behavior? In practice, a researcher would offer evidence that they had controlled for plausible confounders, or at least present a theoretical argument why some confounder doesn’t matter. Note here that “confounding” might be partial: Perhaps gender does cause both drinking and auto accidents, but maybe alcohol also has an independent effect on accident incidence so that the total association of X and Y reflects both true X-Y causality and confounding by C.

Beyond these foundational criteria for causality, knowing something about two common causal patterns (see Figure 1) is also useful. The first of these is commonly called “mediation,” where, given evidence that X does cause Y, interest next rests on what variable(s) M provide the “mediating” mechanism by which causality occurs. So, here, we might theorize that “speed of reaction” time is the variable that alcohol affects and which in turn causes more accidents. Or, perhaps that M variable might instead (or in addition) be “capacity to concentrate.” Determining what variable mediates the X-Y causal relation is often of practical and theoretical interest, and empirical evidence for a particular mediator involves examining what happens if the mediator variable is controlled. Of note, the empirical evidence for a mediator variable is generally the same as for a confounder; however, knowing something about the time order and logical relations among the X, M, and Y enables distinguishing the two patterns.

The second additional causal pattern of interest is termed “moderation” or “interaction.” This pattern describes how the effect of X on Y differs depending on some third variable Z. So, we might suspect alcohol consumption affects accident incidence differently depending on whether food has been consumed, so that alcohol has a big effect among persons with an empty stomach, and much less of an effect among persons who had eaten. More complicated patterns of moderation can exist, a particularly dramatic form of which is when X has a positive effect for certain values of the moderator Z, but a negative effect for other values of Z. Evidence showing that Z moderates the effect of X (phrased otherwise: that X and Z “interact” in their effect on Y) entails (at base) comparing the association of X and Y among individuals having different values of Z, and again, in statistical practice, various methods for doing this exist.

This concludes our brief introduction. For the reader interested in learning more about contemporary concepts in causal inference, Pearl and Mackenzie (2018) offers a popular audience introduction, while Pearl, Glymour, and Jewell 2016) (among many others) provides a more technical starting point.

**References**

Hume, D. (2000). *A treatise of human nature*. Oxford University Press.

Pearl, J., Glymour, M., & Jewell, N. P. (2016). *Causal inference in statistics: A primer*. John Wiley & Sons.

Pearl, J., & Mackenzie, D. (2018). *The book of why: The new science of cause and effect*. Basic Books.
